# Carbamazepine Attenuates Astroglial L-Glutamate Release Induced by Pro-Inflammatory Cytokines via Chronically Activation of Adenosine A_2A_ Receptor

**DOI:** 10.3390/ijms20153727

**Published:** 2019-07-30

**Authors:** Motohiro Okada, Kouji Fukuyama, Takashi Shiroyama, Yuto Ueda

**Affiliations:** Department of Neuropsychiatry, Division of Neuroscience, Graduate School of Medicine, Mie University, Tsu 514-8507, Japan

**Keywords:** carbamazepine, L-glutamate, adenosine receptor, tripartite synaptic transmission, astrocyte

## Abstract

Carbamazepine (CBZ) binds adenosine receptors, but detailed effects of CBZ on astroglial transmission associated with adenosine receptor still need to be clarified. To clarify adenosinergic action of CBZ on astroglial transmission, primary cultured astrocytes were acutely or chronically treated with CBZ, proinflammatory cytokines (interferon γ (IFNγ) and tumor necrosis factor α (TNFα)), and adenosine A2A receptor (A2AR) agonist (CGS21680). IFNγ and TNFα increased basal, adenophostin-A (AdA)-evoked, and 2-amino-3-(3-hydroxy-5-methyl-isoxazol-4-yl)propanoic acid (AMPA)-evoked astroglial L-glutamate releases. In physiological condition, CGS21680 increased basal astroglial L-glutamate release but glutamate transporter inhibition prevented this CGS21680 action. CBZ did not affect basal release, whereas glutamate transporter inhibition generated CBZ-induced glutamate release. Furthermore, AdA-evoked and AMPA-evoked releases were inhibited by CBZ but were unaffected by CGS21680. Contrary to physiological condition, chronic administrations of IFNγ and TNFα enhanced basal, AdA-, and AMPA-evoked releases, whereas IFNγ and TNFα decreased and increased CGS21680-evoked releases via modulation A2AR expression. Both chronic administration of CGS21680 and CBZ suppressed astroglial L-glutamate release responses induced by chronic cytokine exposer. Especifically, chronic administration of CBZ and CGS21680 prevented the reduction and elevation of A2AR expression by respective IFNγ and TNFα. These findings suggest that A2AR agonistic effects of CBZ contribute to chronic prevention of pathomechanisms developments of several neuropsychiatric disorders associated with proinflammatory cytokines.

## 1. Introduction

It has been demonstrated that dysfunctions of glial mechanisms, including self-reinforcing interplay between dysfunctional energy homeostasis, inflammation, and astroglial signaling, play important roles in pathomechanisms of development of several neuropsychiatric disorders, including epilepsy, bipolar disorder, schizophrenia, and chronic pain [1,2,3,4]. Both preclinical and clinical studies have demonstrated astroglial abnormalities in central nervous system of patients with epilepsy and other neuropsychiatric disorders [1,5,6,7]. Specifically, proinflammatory cytokines, tumor necrosis factor α (TNFα), and interferon γ (IFNγ) probably contribute to development of epilepsy (epileptogenesis, ictogenesis, and seizure-related brain damages) [7,8], and several neuropsychiatric disorders [1]. These cytokines enhance astroglial glutamatergic transmission, which is compensated by anticonvulsant, carbamazepine (CBZ), and levetiracetam [8,9,10], as well as antipsychotics, aripiprazole, and clozapine [11,12,13].

CBZ has a wide clinical spectrum against focal epilepsy and trigeminal pain as a first-line medication, and specific subtypes of other neuropsychiatric disorders, bipolar disorder, and schizophrenia [14,15]. The major mechanisms of anticonvulsive action of CBZ has been considered to be inhibition of voltage-gated sodium channels [16], but various pharmacodynamic studies suggest the detailed mechanisms of clinical action of CBZ are more complex with calcium-induced calcium-releasing system [17], glutamate release [18,19], and modulation of voltage-sensitive calcium channels [17,18,19,20,21,22,23]. Indeed, although CBZ inhibits voltage-gated sodium channels, therapeutic-relevant concentration of CBZ increased basal releases of GABA, dopamine, serotonin, and acetylcholine without affecting that of L-glutamate by in vivo microdialysis study [17,19,23,24,25].

Traditionally, the effects of CBZ on adenosine receptor function had been explored, since CBZ binds to adenosine receptors [26,27,28,29,30]. Both adenosine A1 receptor (A1R) agonists and adenosine A2A receptor (A2AR) antagonists can exhibit anticonvulsive actions; however, CBZ is considered an A1R antagonist and an A2AR agonist, since chronic CBZ administration resulted in upregulation of A1R [26] and functional downregulation (or desensitization) of A2AR [27]. Electrophysiological study also demonstrated that therapeutic-relevant concentration of CBZ enhanced hippocampal excitatory postsynaptic current via inhibition of A1R [31]. Additionally, we have already demonstrated that therapeutic-relevant concentration of CBZ increased basal monoamine release [32,33] by inhibition of A1R with enhancement of A2AR activity using in vivo microdialysis [29,30]. Thus, in spite of these efforts, the acute effects of CBZ on adenosine receptor subtypes should be counterproductive to its anticonvulsant activity.

CBZ and levetiracetam suppress the cellular excitability induced by IFNγ and TNFα [8,9,10]. Activation of A2AR, which is dominantly expressed in astrocytes [34], reduced the production of IFNγ and TNFα [35,36] and suppressed proinflammatory responses associated with these cytokines [37,38]. Based on these clinical and preclinical findings, the mechanisms of clinical action of CBZ possibly are involved in the attenuation of proinflammatory cytokines functions via its A2AR agonistic action. The anticonvulsive effects of CBZ are not fully explained by its A1R antagonistic and A2AR agonistic actions in neuron, and the effects of acute and chronic administrations of CBZ on astroglial A2AR have not been clarified. Therefore, to explore the mechanisms of clinical action of CBZ associated with adenosine receptor, the present study determined 1) the effects of acute and chronic administrations of adenosine receptor ligands and CBZ on astroglial L-glutamate release, 2) effects of chronic administration of IFNγ and TNFα on astroglial L-glutamate release, and 3) interactions between adenosine receptor ligands (CGS21680 and CBZ) and proinflammatory cytokines (IFNγ and TNFα) on astroglial L-glutamate release, expression of A2AR, and astroglial glutamate transporter (Slc1a2 and Slc1a3), using primary cultured astrocytes.

## 2. Results

### 2.1. Chronic Effects of IFNγ and TNFα on Basal, AMPA-, and AdA-Evoked Astroglial L-Glutamate Releases (Study 1)

**Figure 1:**
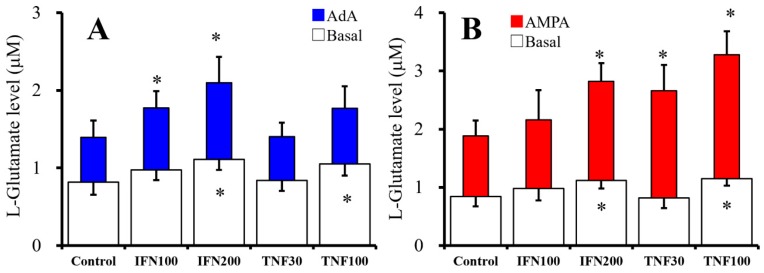
Effects of chronic administration of interferon γ (IFNγ) and tumor necrosis factor α (TNFα) for seven days on basal, 1 μM adenophostin-A (AdA)- and 100 μM 2-amino-3-(3-hydroxy-5-methyl-isoxazol-4-yl)propanoic acid (AMPA)-evoked astroglial L-glutamate releases. Concentration-dependent effects of chronic administration of IFNγ (100 and 200 IU/mL) and TNFα (30 and 100 IU/mL) on basal (opened), 1 μM AdA-evoked (**A**, blue), and 100 μM AMPA-evoked (**B**, red) L-glutamate releases are indicated in Figure 1A and 1B, respectively. Ordinates indicate mean levels of L-glutamate (μM) (*N* = 6). * *p* < 0.05 relative to control by one-way ANOVA with Tukey’s post hoc test.

Both clinical and preclinical studies have indicated that proinflammatory cytokines, including IFNγ and TNFα, play important roles in the development of epilepsy and several neuropsychiatric disorders [1,7]. Indeed, levetiracetam inhibits IFNγ-induced activation of inositol trisphosphate receptor (IP3-R)-associated astroglial transmission of kynurenine pathway [8]. Thus, through screening the target concentration of IFNγ and TNFα, the concentration-dependent effects of chronic administration of IFNγ and TNFα on basal, 1 μM adenophostin-A (AdA)-, and 100 μM 2-amino-3-(3-hydroxy-5-methyl-isoxazol-4-yl)propanoic acid (AMPA)-evoked astroglial L-glutamate release were determined. Cultured astrocytes were incubated in Dulbecco’s modified Eagle’s medium containing 10% fetal calf serum (fDMEM) alone (control) or fDMEM containing IFNγ (100 and 200 IU/mL) or TNFα (30 and 100 IU/mL) for 7 days, from after 21 days of culture (DIV21) to DIV28. After wash-out at DIV28, astrocytes were incubated in artificial cerebrospinal fluid (ACSF) containing the same cytokine for 30 min, and ACSF was collected for analysis of basal release. After collection for basal release sample, the astrocytes were incubated in the same ACSF containing AMPA (100 μM) or AdA (1 μM) for 30 min (Figure 1).

Both chronic administrations of IFNγ (100 and 200 IU/mL) [F(2,15) = 6.3 (*p* < 0.05)] and TNFα (30 and 100 IU/mL) [F(2,15) = 4.6 (*p* < 0.05)] increased basal L-glutamate release in a concentration-dependent manner (Figure 1A,B). AdA (1 μM) and AMPA (100 μM) increased L-glutamate release (Figure 1A,B). AdA-evoked release was also increased by chronic administration of IFNγ in a concentration-dependent manner [F(2,15) = 9.8 (*p* < 0.05)] but was not affected by TNFα (Figure 1A,B). In contrast with AdA-evoked release, AMPA-evoked release was increased, in a concentration-dependent manner, by chronic administration of IFNγ [F(2,15) = 12.1 (*p* < 0.05)] and TNFα [F(2,15) = 24.7 (*p* < 0.05)] (Figure 1A,B).

Chronic administration of both IFNγ and TNFα increased basal astroglial L-glutamate release concentration-dependently (Figure 1A,B). Contrary to basal release, chronic IFNγ administration enhanced predominantly AdA-evoked rather than AMPA-evoked L-glutamate release, whereas chronic TNFα administration enhanced predominantly AMPA-evoked rather than AdA-evoked L-glutamate release (Figure 1A,B).

### 2.2. Acute and Chronic Effects of Therapeutic-Relevant Concentration of CBZ on Astroglial Releases of L-Glutamate (Study 2)

The minimum CBZ plasma concentration that is effective against maximal electroshock seizures in rats ranges from 17 to 42 μM [39]. Therefore, 10 μM and 20 μM CBZ were administrated as a lower than and therapeutic-relevant concentrations.

**Figure 2:**
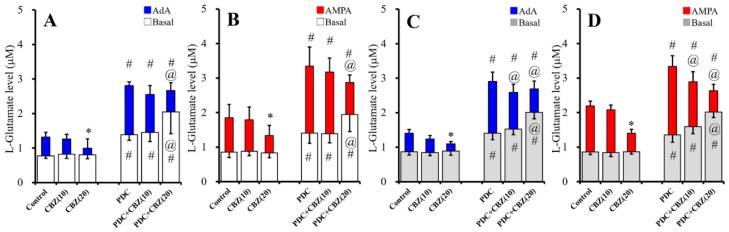
Effects of acute (**A**,**B**) and chronic (**C**,**D**) administrations of carbamazepine (CBZ) (10 and 20 μM), under the glutamate transporter functional and blockade by trans-4-carboxy-L-proline (PDC) (50 μM), on basal (opened and gray), 1 μM AdA-evoked (blue), and 100 μM AMPA-evoked (red) L-glutamate releases are represented in Figure 2. Ordinates indicate mean levels of L-glutamate (μM) (*N* = 6). * *p* < 0.05; relative to control, @ *p* < 0.05; relative to PDC, and # *p* < 0.05 relative to non PDC (control vs. PDC or CBZ vs. PDC + CBZ) by two-way ANOVA with Tukey’s post hoc test.

To study the effects of acute administration of CBZ (10 and 20 μM) on astroglial L-glutamate release, cultured astrocytes collected on DIV28 were washed-out with ACSF, then incubated on translucent PET membranes for 30 min in ACSF alone (control) or ACSF containing glutamate transporter inhibitor, trans-4-carboxy-L-proline (PDC: 50 μM), CBZ (10 or 20 µM), or PDC (50 µM) plus CBZ (10 or 20 µM). After collecting the basal release samples, the astrocytes were incubated on translucent PET membranes for 30 min in ACSF containing the same agent with AMPA (100 μM) or AdA (1 μM) (Figure 2A,B). Acute administration of CBZ (10 and 20 μM) did not affect basal L-glutamate release (Figure 2A,B); however, when the L-glutamate transporter was inhibited by PDC (50 μM), which increased the basal level of L-glutamate release (Figure 2A,B), acute administration of 10 and 20 μM CBZ did not affect and increased basal L-glutamate release, respectively [F_CBZ_(2,35) = 4.7 (*p* < 0.05), F_PDC_(1,35) = 70.8 (*p* < 0.05), F_PDC*CBZ_(2,35) = 4.4 (*p* < 0.05)] (Figure 2A,B). In contrast, 1 μM AdA-evoked L-glutamate release was not affected and decreased by acute administration of 10 and 20 μM CBZ, respectively [F_CBZ_(2,35) = 21.1 (*p* < 0.05), F_PDC_(1,35) = 80.8 (*p* < 0.05), F_PDC*CBZ_(2,35) = 3.1 (*p* > 0.05)] (Figure 2A). Further, 100 μM AMPA-evoked L-glutamate release was also not affected and decreased by acute administration of 10 and 20 μM CBZ, respectively [F_CBZ_(2,35) = 29.7 (*p* < 0.05), F_PDC_(1,35) = 75.5 (*p* < 0.05), F_PDC*CBZ_(2,35) = 3.6 (*p* < 0.05)] (Figure 2B).

To study the effects of chronic administration of CBZ (10 and 20 μM) for seven days on astroglial L-glutamate release, from DIV21 to DIV28, cultured astrocytes were incubated in fDMEM alone (control) or fDMEM containing CBZ (10 or 20 μM). After wash-out by ACSF at DIV28, cultured astrocytes were incubated in ACSF alone (control) or ACSF containing CBZ for 30 min. ACSF was collected for analysis of basal release. After collection for basal release, astrocytes were incubated in ACSF containing the same agent with 1 μM AdA or 100 μM AMPA for 30 min (Figure 2C,D). Chronic administration of CBZ (10 and 20 μM) did not affect basal L-glutamate release (Figure 2C,D); however, when the L-glutamate transporter was inhibited by PDC (50 μM), which increased the basal level of L-glutamate release (Figure 2C,D), chronic administration of 10 and 20 μM CBZ did not affect and increased basal L-glutamate release, respectively [F_CBZ_(2,35) = 16.0 (*p* < 0.05), F_PDC_(1,35) = 255.5 (*p* < 0.05), F_PDC*CBZ_(2,35) = 13.2 (*p* < 0.05)] (Figure 2C,D). In contrast, chronic administration of CBZ (10 and 20 μM) decreased AdA-evoked [F_CBZ_(2,35) = 124.8 (*p* < 0.05), F_PDC_(1,35) = 557.9 (*p* < 0.05), F_PDC*CBZ_(2,35) = 24.3 (*p* < 0.05)] and AMPA-evoked [F_CBZ_(2,35) = 374.6 (*p* < 0.05), F_PDC_(1,35) = 71.9 (*p* < 0.05), F_PDC*CBZ_(2,35) = 27.5 (*p* < 0.05)] L-glutamate release (Figure 2C,D).

Both acute and chronic administration of therapeutic-relevant concentration of CBZ inhibited AdA-evoked and AMPA-evoked L-glutamate release without affecting basal release under the condition of astroglial glutamate transporter functional; however, under the astroglial glutamate transporter blockade, acute and chronic administration of therapeutic-relevant concentration of CBZ increased basal L-glutamate release, but inhibited AdA-evoked and AMPA-evoked releases (Figure 2).

### 2.3. Acute Effects of Adenosine Receptor Agents on Astroglial L-Glutamate Release (Study 3)

**Figure 3:**
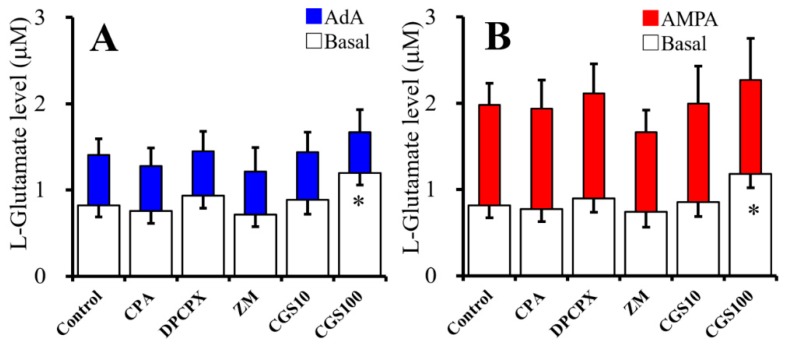
Acute effects of adenosine receptor agents on astroglial L-glutamate release. Figure 3A,B indicates effects of acute administration of N6-cyclopentyladenosine (CPA) (10 nM), 8-cyclopentyl-1,3-dipropylxanthine (DPCPX) (100 nM), ZM241385 (ZM: 100 nM), and CGS21680 (CGS: 10 and 100 nM) on basal (opened), 1 μM AdA-evoked (**A**, blue), and 100 μM AMPA-evoked (**B**, red) L-glutamate release, respectively. Ordinates indicate mean levels of L-glutamate (μM) (*N* = 6). * *p* < 0.05; relative to control by one-way ANOVA with Tukey’s post hoc test.

In order to clarify the predominant functional adenosine receptor subtypes on astroglial L-glutamate release, cultured astrocytes collected at DIV28 were washed-out with ACSF, then incubated on translucent PET membranes for 30 min in ACSF alone (control) or ACSF containing A1R agonist, N6-cyclopentyladenosine (CPA: 10 nM), A1R antagonist; 8-cyclopentyl-1,3-dipropylxanthine (DPCPX: 100 nM), A2AR agonist, CGS21680 (10 or 100 nM), or A2AR antagonist, ZM241385 (100 nM). After collecting basal release samples, the astrocytes were incubated on the translucent PET membranes for 30 min in ACSF containing the same agent with 100 µM AMPA (AMPA-evoked release) or 1 µM AdA (AdA-evoked release) (Figure 3A,B).

Neither CPA (10 nM), DPCPX (100 nM) nor ZM241385 (100 nM) affected basal, 1 μM AdA-, or 100 μM AMPA-evoked astroglial L-glutamate releases (Figure 3A,B). In contrast, CGS21680 (10 and 100 nM) increased basal L-glutamate release in a concentration-dependent manner [F(2,15) = 12.0 (*p* < 0.05)]; however, neither 10 nM nor 100 nM CGS21680 affected AdA- and AMPA-evoked L-glutamate release (Figure 3A,B). Therefore, A2AR phasically activates basal astroglial L-glutamate release, but A1R does not affect (Figure 3A,B). Contrary to basal release, neither IP3-R- and AMPA-R-associated astroglial L-glutamate release were acutely affected by A2AR (Figure 3A,B).

### 2.4. Effects of ZM241385 on CGS21680- and CBZ-evoked astroglial L-glutamate releases (Study 4)

**Figure 4:**
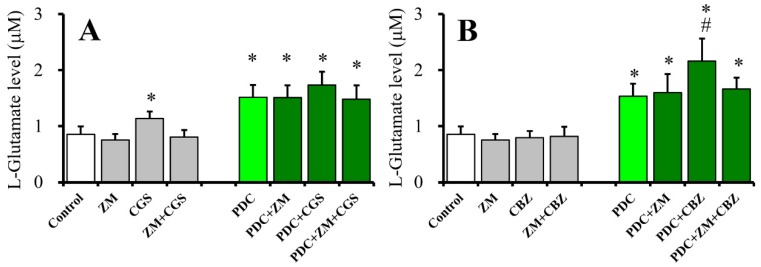
Effects of 100 nM ZM241285 (A2AR antagonist) on 100 nM CGS21680-evoked (**A**) and 20 μM CBZ-evoked (**B**) astroglial L-glutamate releases. Acute effects of A2AR antagonist (ZM: 100 nM) on 100 nM CGS21680-evoked L-glutamate release under the glutamate transporter functional (opened and gray) and blockade (green) are indicated in Figure 4A. Acute effects of A2AR antagonist on 20 μM CBZ-evoked L-glutamate release under the glutamate transporter functional (opened and gray) and blockade (green) are indicated in Figure 4B. Ordinates indicate mean levels of L-glutamate (μM) (*N* = 6). * *p* < 0.05 relative to control and # *p* < 0.05 relative to PDC by two-way ANOVA with Tukey’s post hoc test.

In order to study the mechanisms of the stimulatory effects of 100 nM CGS21680 and 20 μM CBZ on basal L-glutamate release, after wash-out at DIV28, cultured astrocytes were incubated for 30 min in ACSF alone (control) or ACSF containing ZM241385 (100 nM), CGS201680 (100 nM), CBZ (20 μM), ZM241385 (100 nM) plus CGS201680 (100 nM), or CBZ (20 μM) (Figure 4A,B). ZM241385 (100 nM) alone did not affect basal L-glutamate release (Figure 4A,B), but inhibited 100 nM CGS21680-evoked L-glutamate release [F_ZM_(1,20) = 18.6 (*p* < 0.05), F_CGS_(1,20) = 12.0 (*p* < 0.05), F_ZM*CGS_(1,20) = 5.4 (*p* < 0.05)] (Figure 4A). Therefore, elevation of basal L-glutamate release induced by CGS21680 is, at least partially, generated by activation of A2AR. Contrary to CGS21680, CBZ (20 μM) did not affect basal L-glutamate release (Figure 4B).

To clarify the mechanisms of CBZ-induced astroglial L-glutamate release under the glutamate transporter blockade, after wash-out at DIV28, cultured astrocytes were incubated for 30 min in ACSF alone (control) or ACSF containing PDC (50 μM) with ZM241385 (100 nM), CGS21680 (100 nM), CBZ (20 μM), ZM241385 (100 nM) plus CGS21680 (100 nM), or CBZ (20 μM) (Figure 4A,B). Neither ZM241385 (100 nM), CGS21680 (100 nM), nor ZM241385 (100 nM) plus CGS21680 (100 nM) affected basal L-glutamate release, under the glutamate transporter blockade (Figure 4A). Contrary to CGS21680, therapeutic-relevant concentration of CBZ (20 μM) increased L-glutamate release (CBZ-evoked L-glutamate release), under the glutamate transporter blockade. ZM241385 (100 nM) alone did not affect basal L-glutamate release but inhibited CBZ-evoked L-glutamate release [F_ZM_(1,20) = 3.1 (*p* > 0.5), F_CBZ_(1,20) = 8.0 (*p* < 0.05), F_ZM*CBZ_(1,20) = 5.1 (*p* < 0.05)] (Figure 4B). These results suggest the possible mechanisms that both CGS21680-evoked and CBZ-induced L-glutamate releases are generated by A2AR activation, since A2AR antagonist prevent these L-glutamate releases. However, CGS21680-evoked release is probably modulated by inhibition of astroglial glutamate transporter, but CBZ-evoked release is another mechanism regarding to astroglial glutamate transporter.

### 2.5. Interaction Between Chronic Cytokines Administration and Acute CBZ Administration on Basal, AdA-, and AMPA-Evoked Releases of L-Glutamate (Study 5)

**Figure 5:**
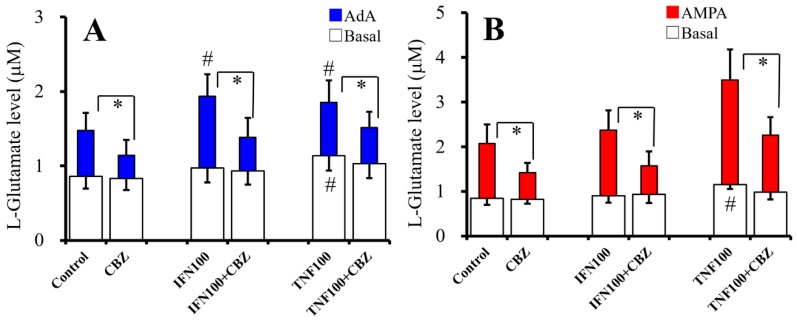
Interaction between chronic cytokines administration and acute CBZ administration on basal, AdA-, and AMPA-evoked releases of L-glutamate. Interaction between chronic cytokines (100 IU/mL IFNγ and TNFα) administration and acute CBZ (20 μM) administration on basal (opened) and 1 μM AdA-evoked (**A**, blue) L-glutamate release are indicated in Figure 5A. Interaction between chronic cytokines (100 IU/mL IFNγ and TNFα) administration and acute CBZ (20 μM) administration on basal (opened) and 100 μM AMPA-evoked (**B**, red) L-glutamate release are indicated in Figure 5B. Ordinates indicate mean levels of L-glutamate (μM) (*N* = 6). * *p* < 0.05 relative to non-CBZ and # *p* < 0.05 relative to control by two-way ANOVA with Tukey’s post hoc test.

To study the effects of chronic administration of cytokines (100 IU/mL IFNγ and TNFα) and acute administration of therapeutic-relevant concentration of CBZ (20 μM) on basal and 1 μM AdA- and 100 μM AMPA-evoked L-glutamate release, cultured astrocytes were incubated in fDMEM alone (control) or fDMEM containing with IFNγ or TNFα for seven days (from DIV21 to DIV28). After wash-out at DIV28, astrocytes were incubated in ACSF alone (control) or ACSF containing the same cytokine with CBZ for 30 min, and ACSF was collected for analysis of basal release. After collection for basal release, astrocytes were incubated in ACSF containing the same cytokine with 1 μM AdA or 100 μM AMPA for 30 min (Figure 5).

Acute administration of CBZ (20 μM) inhibited the effects of chronic administration of IFNγ (100 IU/mL) and TNFα (100 IU/mL) on basal [F_CBZ_(1,30) = 0.9 (*p* > 0.1), F_Cytokine_(2,30) = 5.2 (*p* < 0.05), F_CBZ*cytokine_(2,30) = 5.5 (*P* < 0.05)] and 1 μM AdA-evoked [F_CBZ_(1,30) = 92.5 (*p* < 0.05), F_Cytokine_(2,30) = 8.1 (*p* < 0.05), F_CBZ*cytokine_(2,30) = 5.2 (*p* < 0.05)] L-glutamate releases (Figure 5A). Chronic IFNγ administration increased AdA-evoked L-glutamate release, and CBZ inhibited IFNγ-induced elevation of AdA-evoked L-glutamate release (Figure 5A). Chronic TNFα administration increased basal and AdA-evoked L-glutamate releases, and CBZ inhibited TNFα-induced elevation of both basal and AdA-evoked release (Figure 5A). Acute administration of CBZ (20 μM) inhibited the effects of chronic administrations of IFNγ (100 IU/mL) and TNFα (100 IU/mL) on 100 μM AMPA-evoked L-glutamate release [F_CBZ_(1,30) = 52.3 (*p* < 0.05), F_Cytokine_(2,30) = 22.8 (*p* < 0.05), F_CBZ*cytokine_(2,30) = 4.1 (*p* < 0.05)] (Figure 5B). Chronic TNFα administration increased basal and AMPA-evoked L-glutamate release, and CBZ inhibited TNFα-induced elevation of both basal and AMPA-evoked release (Figure 5B). Thus, acute administration of therapeutic-relevant concentration of CBZ suppresses basal, AdA-, and AMPA-evoked L-glutamate release of chronic expose to IFNγ and TNFα.

### 2.6. Interaction Between Chronic Administration of Cytokines and CBZ on Basal AdA-, and AMPA-Evoked L-Glutamate Releases (Study 6)

**Figure 6:**
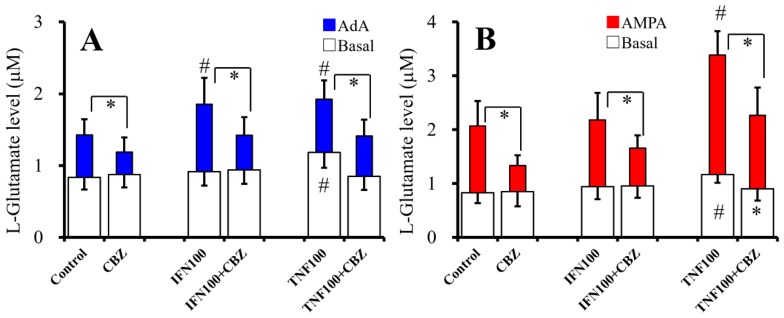
Interaction between chronic administration of cytokines and CBZ on basal, AdA-, and AMPA-evoked L-glutamate releases. Effects of chronic administration of IFNγ (100 IU/mL), TNFα (100 IU/mL), and CBZ (20 μM) on basal (opened) and 1 μM AdA-evoked (**A**, blue) L-glutamate release are indicated in Figure 6A. Effects of chronic administration of IFNγ (100 IU/mL), TNFα (100 IU/mL) with CBZ (20 μM) on basal (opened), and 100 μM AMPA-evoked (**B**, red) L-glutamate release are indicated in Figure 6B. Ordinates indicate mean levels of L-glutamate (μM) (*N* = 6). * *p* < 0.05 relative to non-CBZ and # *p* < 0.05 relative to control by two-way ANOVA with Tukey’s post hoc test.

To study the effects of chronic administration of cytokines (100 IU/mL IFNγ and TNFα) and CBZ (20 μM) on basal, 1 μM AdA-, and 100 μM AMPA-evoked release of L-glutamate, cultured astrocytes were incubated in fDMEM alone (control) or fDMEM containing IFNγ, TNFα, CBZ plus IFNγ, or TNFα for seven days (from DIV21 to DIV28). After wash-out at DIV28, astrocytes were incubated in ACSF containing the same agents for 30 min and collected for analysis of basal release. After collection for basal release, astrocytes were incubated in ACSF containing the same agents with 1 μM AdA or 100 μM AMPA for 30 min (Figure 6).

Chronic administration of therapeutic-relevant concentration of CBZ (20 μM) inhibited the effects of chronic administration of IFNγ (100 IU/mL) and TNFα (100 IU/mL) on basal [F_CBZ_(1,30) = 2.0 (*p* > 0.1), F_cytokine_(2,30) = 2.1 (*p* > 0.1), F_CBZ*cytokine_(2,30) = 3.6 (*p* < 0.05)] and 1 μM AdA-evoked [F_CBZ_(1,30) = 55.0 (*p* < 0.05), F_cytokine_(2,30) = 14.6 (*p* < 0.05), F_CBZ*cytokine_(2,30) = 3.8 (*p* < 0.05)] L-glutamate release (Figure 6A). Chronic IFNγ administration increased AdA-evoked release, and chronic CBZ administration inhibited IFNγ-induced elevation of AdA-evoked release (Figure 6A). Chronic TNFα administration increased basal and AdA-evoked L-glutamate release, and chronic CBZ administration inhibited TNFα-induced elevation of both basal and AdA-evoked release (Figure 6A).

Chronic administration of CBZ (20 μM) inhibited the effects of chronic administration of IFNγ (100 IU/mL) and TNFα (100 IU/mL) on 100 μM AMPA-evoked L-glutamate release [F_CBZ_(1,30) = 64.0 (*p* < 0.05), F_cytokine_(2,30) = 42.7 (*p* < 0.05), F_CBZ*cytokine_(2,30) = 8.2 (*p* < 0.05)] (Figure 6B). Chronic TNFα administration increased basal and AMPA-evoked release, and chronic CBZ administration inhibited TNFα-induced elevation of both basal and AMPA-evoked release (Figure 6B). Taken together with the results in Study 5 and Study 6, both acute and chronic administrations of therapeutic-relevant concentration of CBZ suppresses basal, AdA-, and AMPA-evoked L-glutamate release of chronic expose to IFNγ and TNFα.

### 2.7. Interaction Between Chronic Administration of Cytokines and CGS21680 on Basal, AdA-, and AMPA-Evoked Releases of L-Glutamate (Study 7) 

**Figure 7:**
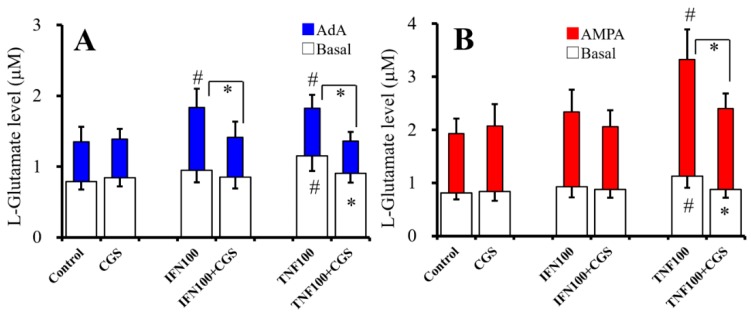
Interaction between chronic administration of cytokines and CGS21680 on basal, AdA-, and AMPA-evoked releases of L-glutamate. Interaction between effects of chronic administration of IFNγ (100 IU/mL), TNFα (100 IU/mL), and CGS21680 (CGS: 10 nM) on basal (opened) and 1 μM AdA-evoked (**A**, blue) L-glutamate release are indicated in Figure 7A. Interaction between effects of chronic administration of IFNγ (100 IU/mL), TNFα (100 IU/mL), and CGS21680 (10 nM) on basal (opened) and 100 μM AMPA-evoked (**B**, red) L-glutamate release are indicated in Figure 7B. Ordinates indicate mean levels of L-glutamate (μM) (*N* = 6). * *p* < 0.05 relative to non-CGS and # *p* < 0.05 relative to control by two-way ANOVA with Tukey’s post hoc test.

To study the effects of chronic administration of cytokines (100 IU/mL IFNγ and TNFα) and sub-effective concentration of CGS21680 (10 nM) on basal, 1 μM AdA-, and 100 μM AMPA-evoked L-glutamate releases, cultured astrocytes were incubated in fDMEM alone (control) or fDMEM containing IFNγ, TNFα, 10 nM CGS21680, CGS21680 plus IFNγ, or TNFα for seven days (from DIV21 to DIV28). After wash-out at DIV28, astrocytes were incubated in ACSF containing the same agents for 30 min, and ACSF was collected for analysis of basal release. After collection for basal release, astrocytes were incubated in ACSF containing the same agents with 1 μM AdA or 100 μM AMPA for 30 min (Figure 7).

Chronic administration of CGS21680 (10 nM) did not affect basal, 1 μM AdA-, or 100 μM AMPA-evoked L-glutamate release (Figure 7A,B). Chronic administration of IFNγ (100 IU/mL) did not affect basal or 100 μM AMPA-evoked L-glutamate release but increased 1 μM AdA-evoked L-glutamate release (Figure 7A,B), whereas chronic administration of CGS21680 inhibited the stimulatory effects of IFNγ on AdA-evoked L-glutamate release [F_CGS_(1,20) = 12.6 (*p* > 0.05), F_IFN_(1,20) = 13.2 (*p* < 0.05), F_CGS*IFN_(1,20) = 11.0 (*p* < 0.05)] (Figure 7A). Chronic administration of TNFα (100 IU/mL) increased basal, AdA-, and AMPA-evoked L-glutamate release (Figure 7A,B). Chronic administration of CGS21680 (10 nM) inhibited the stimulatory effects of chronic administration of TNFα on basal [F_CGS_(1,20) = 2.7 (*p* > 0.5), F_TNF_(1,20) = 12.0 (*p* < 0.05), F_CGS*TNF_(1,20) = 6.0 (*p* < 0.05)], AdA-evoked [F_CGS_(1,20) = 6.2 (*p* < 0.05), F_TNF_(1,20) = 0.1 (*p* > 0.5), F_CGS*TNF_(1,20) = 5.1 (*p* < 0.05)], and AMPA-evoked (F_CGS_(1,20) = 5.4 (*P* < 0.05), F_TNF_(1,20) = 33.0 (*P* < 0.05), F_CGS*TNF_(1,20) = 11.0 (*P* < 0.05)) L-glutamate release (Figure 7A,B).

In physiological condition, neither acute nor chronic administration of 10 nM CGS21680 affected basal, AdA-, and AMPA-evoked L-glutamate release; however, chronic administration of 10 nM CGS21680 suppresses the stimulatory effects of IFNγ and TNFα on basal, AdA-evoked, and AMPA-evoked release. These discrepancies the effects of sub-effective concentration of CGS21680 between physiological and pathological (chronic exposure to cytokines) conditions on astroglial L-glutamate release suggest that chronic activation of A2AR probably suppresses the astroglial proinflammatory responses.

### 2.8. Interaction Between Chronic Administration of Cytokines, CBZ, and CGS21680 on CGS21680-Evoked L-Glutamate Release (Study 8)

**Figure 8:**
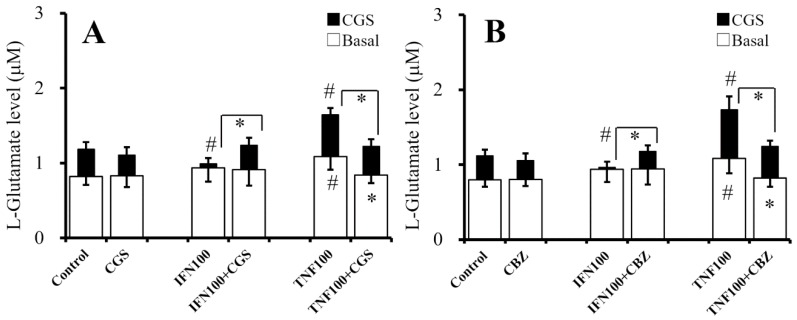
Interaction between chronic administration of cytokines, sub-effective concentration of CGS21680 (10 nM), and therapeutic-relevant concentration of CBZ (20 μM) on 100 nM CGS21680-evoked L-glutamate release. Effects of chronic administration of IFNγ (100 IU/mL), TNFα (100 IU/mL), and CGS (10 nM) on basal (opened) and 100 nM CGS-evoked (closed) releases of L-glutamate from cultured astrocytes are indicated in Figure 8A. Effects of chronic administration of IFNγ (100 IU/mL), TNFα (100 IU/mL), and CBZ (20 μM) on basal (opened) and 100 nM CGS-evoked (closed) releases of L-glutamate from cultured astrocytes are indicated in Figure 8B. Ordinates indicate mean levels of L-glutamate (μM) (*N* = 6). * *p* < 0.05 relative to non-CGS (**A**) or non-CBZ (**B**) and # *p* < 0.05 relative to control by two-way ANOVA with Tukey’s post hoc test.

To study the effects of chronic administration of cytokines (100 IU/mL IFNγ and TNFα) and sub-effective concentration of CGS21680 (10 nM) on effective concentration (100 nM) of CGS21680-evoked L-glutamate release, cultured astrocytes were incubated in fDMEM alone (control) or fDMEM containing IFNγ, TNFα, CGS21680 (10 nM), CGS21680 (10 nM) plus IFNγ, or TNFα for seven days (from DIV21 to DIV28). After wash-out at DIV28, the astrocytes were incubated in ACSF containing the same agents for 30 min and collected for analysis of basal release. After collection for basal release, astrocytes were incubated in ACSF containing the same agents with 100 nM CGS21680 for 30 min (Figure 8).

The interaction between chronic administration of cytokines and sub-effective CGS21680 (10 nM) on 100 nM CGS21680-evoked L-glutamate release was detectable [F_CGS_(1,30) = 2.6 (*p* > 0.05), F_cytokine_(2,30) = 3.3 (*p* < 0.05), F_CGS*cytokine_(2,30) = 3.4 (*p* < 0.05)] (Figure 8A). Chronic administration of IFNγ (100 IU/mL) did not affect basal L-glutamate release, but it did inhibit 100 nM CGS21680-evoked L-glutamate release (Figure 8A). Chronic administration of sub-effective concentration of CGS21680 (10 nM) did not affect basal or 100 nM CGS21680-evoked L-glutamate release but prevented the inhibitory effects of chronic IFNγ administration on CGS21680-evoked release (Figure 8A). Chronic administration of TNFα (100 IU/mL) increased basal and 100 nM CGS21680-evoked L-glutamate release, whereas chronic administration of CGS21680 (10 nM) inhibited the stimulatory effects of chronic TNFα administration on basal and CGS21680-evoked L-glutamate releases (Figure 8A).

To study the effects of chronic administration of cytokines (100 IU/mL IFNγ and TNFα) and CBZ (20 μM) on 100 nM CGS21680-induced L-glutamate release, cultured astrocytes were incubated in fDMEM alone (control) or fDMEM containing IFNγ, TNFα, CBZ, CBZ plus IFNγ, or TNFα for seven days (from DIV21 to DIV28). After wash-out at DIV28, the astrocytes were incubated in ACSF containing the same agents for 30 min and collected for analysis of basal release. After collection for basal release, astrocytes were incubated in ACSF containing the same agents with 100 nM CGS21680 for 30 min (Figure 8B).

The interaction between chronic administration of cytokines and therapeutic-relevant concentration of CBZ (20 μM) on 100 nM CGS21680-induced L-glutamate release was detectable [F_CBZ_(1,30) = 0.1 (*p* > 0.05), F_cytokine_(2,30) = 16.2 (*p* < 0.05), F_CBZ*cytokine_(2,30) = 11.3 (*p* < 0.05)] (Figure 8B). Chronic administration of IFNγ (100 IU/mL) did not affect basal L-glutamate release, but it did inhibit 100 nM CGS21680-induced L-glutamate release (Figure 8B). Chronic administration of therapeutic-relevant concentration of CBZ (20 μM) did not affect basal or 100 nM CGS21680-evoked L-glutamate release but prevented the inhibitory effects of chronic IFNγ administration on CGS21680-evoked release (Figure 8B). Chronic administration of TNFα (100 IU/mL) increased basal and CGS21680-evoked L-glutamate release, whereas chronic administration of CBZ (20 μM) inhibited the stimulatory effects of chronic TNFα administration on basal and CGS21680-evoked L-glutamate releases (Figure 8B).

Both chronic administration of IFNγ and TNFα enhance basal, AdA-, and AMPA-evoked release; A2AR-associated basal L-glutamate release (CGS21680-evoked release) was inhibited and activated by chronic administration of IFNγ and TNFα, respectively. In physiological condition, neither chronic administration of sub-effective concentration of CGS21680 (10 nM) nor therapeutic-relevant concentration of CBZ (20 μM) affected 100 nM CGS21680-evoked L-glutamate release but suppresses the inhibitory effect of IFNγ and stimulatory effects of TNFα on CGS21680-evoked release. These contradictive actions of chronic administrations of CGS21680, CBZ, and cytokines suggest that chronic activation of A2AR also probably suppresses the astroglial proinflammatory responses.

### 2.9. Interaction Between Chronic Administration of Cytokines, CBZ, and CGS21680 on mRNA Expression of A2AR and Glutamate Transporters

The results of Study 7 and Study 8 indicate the chronic administrations of CGS21680, CBZ, and cytokines drastically changes the regulation mechanism of astroglial L-glutamate release, including A2AR expression. To explore the mechanisms of unexpected effects of chronic administration (for seven days) of cytokines (100 IU/mL IFNγ and TNFα), sub-effective concentration of CGS21680 (10 nM), and therapeutic-relevant concentration of CBZ (20 μM) on 100 nM CGS201680-evoked L-glutamate release, the effects of chronic administrations of these agents on mRNA expression of A2AR (*Adora2a*) and astroglial glutamate transporter (*Slc1a2* and *Slc1a3*) in cultured astrocytes were determined.

**Figure 9:**
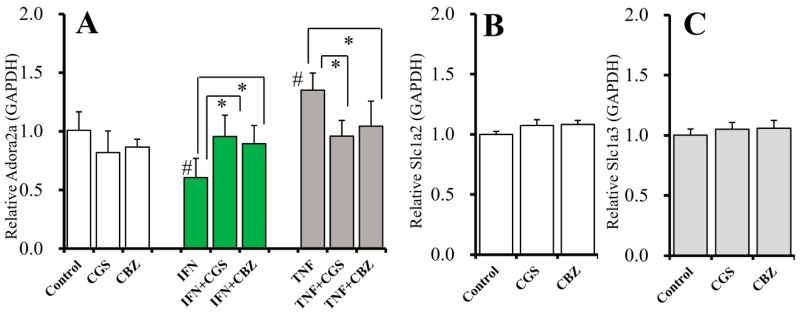
Interaction between chronic administration of IFNγ, TNFα, CGS21680, and CBZ on mRNA expression of A2AR (*Adora2a*) and astroglial glutamate transporters (*Slc1a2* and *Slc1a3*). (**A**) Effects of chronic administration of IFNγ (100 IU/mL), TNFα (100 IU/mL), CGS21680 (10 nM), and CBZ (20 μM) on Adora2a expression in astrocytes. (**B**,**C**) Effects of chronic administration of CGS21680 (10 nM) and CBZ (20 μM) on expression of *Slc1a2* and *Slc1a3* in astrocytes, respectively. Ordinates indicate the relative mRNA expression to *GAPDH* (*N* = 6). * *p* < 0.05 relative to non CGS21680 or CBZ and # *p* < 0.05 relative to control by two-way ANOVA with Tukey’s post hoc test.

The interaction among chronic administration of cytokines, sub-effective CGS21680 (10 nM), and therapeutic-relevant concentration of CBZ (20 μM) on *Adora2a* expression in astrocytes was detectable [F_agents_(2,45) = 1.1 (*p* > 0.05), F_cytokine_(2,45) = 16.8 (*p* < 0.05), F_gents*cytokine_(4,45) = 9.6 (*p* < 0.05)] (Figure 9A). Chronic administration of IFNγ (100 IU/mL) and TNFα (100 IU/mL) decreased and increased *Adora2a* expression in astrocytes, respectively (Figure 9A). The inhibitory effect of IFNγ on *Adora2a* expression was inhibited by chronic administration of CGS21680 and CBZ (Figure 9A). The stimulatory effect of TNFα on *Adora2a* expression was also inhibited by chronic administration of CGS21680 and CBZ (Figure 9A). Contrary to *Adora2a*, neither chronic administration of CGS21680 (10 nM) nor CBZ (20 μM) affected the mRNA expression of astroglial glutamate transporters (*Slc1a2* and *Slc1a3*) in astrocytes (Figure 9B,C).

The inhibitory effect of IFNγ and stimulatory effect of TNFα on CGS21680-evoked release are, at least partially, generated by the decreased and increased expression of astroglial A2AR expression, respectively. Chronic administration of CGS21680 and CBZ prevent these contradictive actions induced by IFNγ and TNFα without affecting astroglial transporter expression.

## 3. Discussion

**Figure 10:**
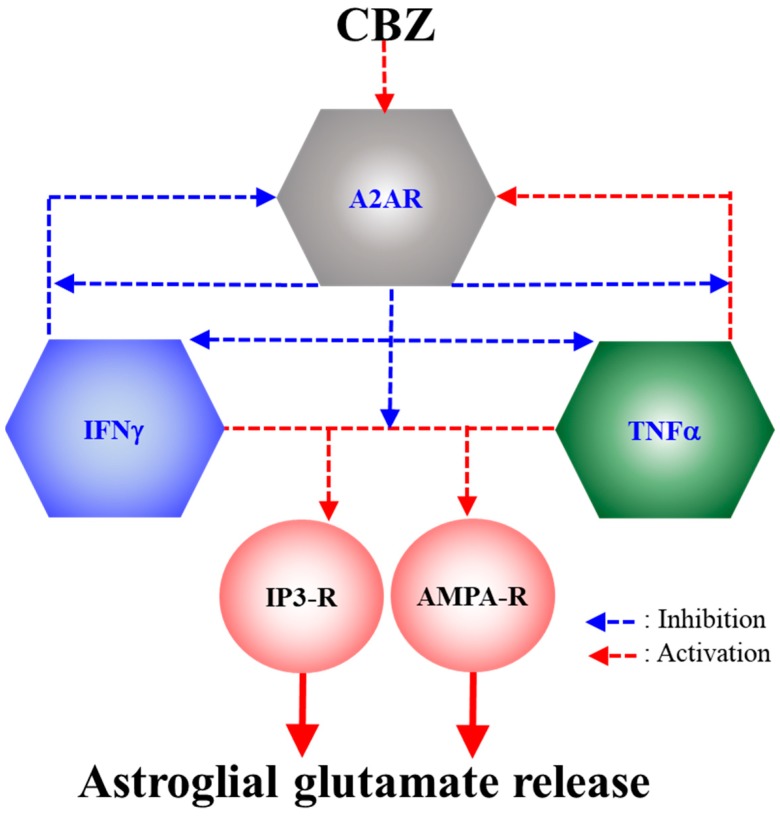
Our proposed hypothesis for the mechanisms of CBZ on astroglial glutamatergic transmission associated with A2AR. Chronic exposure to IFNγ and TNFα suppresses and enhances astroglial A2AR expression, respectively; however, chronic activation of astroglial A2AR inhibits the expressions and responses of IFNγ and TNFα. Both IFNγ and TNFα enhance astroglial transmission via activations of IP3-R and AMPA-R. Chronic administration of CBZ suppresses the stimulatory effects of IFNγ and TNFα on astroglial glutamatergic transmission via its A2AR agonistic action.

The results in this study regarding effects of acute and chronic administrations of therapeutic-relevant concentration of CBZ (20 μM) and CGS21680 on astroglial glutamatergic transmission are summarized in Table 1 and Table 2. Additionally, the candidate mechanism of CBZ on astroglial transmission based on the demonstrations in this study is also summarized in Figure 10.

### 3.1. Astroglial L-Glutamate Release Mechanism Associated with A2AR 

We have already determined the various regulation mechanisms of astroglial L-glutamate release, including through exocytosis [8,12,40], hemichannel [11], and transporter [2,41], using primary cultured astrocytes. Especially, the pharmacological profiles of astroglial L-glutamate exocytosis are tetanus-toxin-sensitive, fluorocitrate-sensitive, calcium-dependent, but tetrodotoxin-insensitive [8,12,40,42].

Extracellular adenosine is derived from the rapid adenosine triphosphate hydrolysis released from both neurons and astrocytes [43]. Various studies demonstrated that A1R decreased the release of several neurotransmitters in vivo [29,30,44,45], probably via voltage-sensitive Ca^2+^ channel inhibition [46] and opening of K^+^ channels [47]. Contrary to A1R, activation of A2AR increased neurotransmitter release [29,30,44,45] via reduced pre-synaptic inhibition associated with A1R [29,30,44,45,48]. Our previous studies using microdialysis demonstrated the stimulatory effects of A2AR could not be observed in the presence of functional A1R, but A2AR activation under the condition of A1R blockade increased monoamine release [29,30,44,45]. Based on our previous results, we concluded that A1R and A2AR are inhibitory and stimulatory neuro-functional receptors, respectively.

The results of this study clearly show that the mechanisms of L-glutamate release of neurons and astrocytes differ, since basal astroglial L-glutamate release is predominantly regulated by astroglial A2AR rather than A1R. Neither A1R agonist nor antagonist affected astroglial L-glutamate release; however, acute administration of effective concentration of A2AR agonist (CGS21680: 100 nM) increased L-glutamate release from primary cultured astrocytes, irrespective of whether A1R was functional and blockade. Additionally, acute administration of an A2AR antagonist (ZM241385) alone did not affect astroglial L-glutamate release but did inhibit 100 nM CGS21680-evoked astroglial L-glutamate release. Therefore, elevating astroglial transmitter release by activating A2AR is induced by A2AR phasic activation-induced release. These stimulatory effects of CGS21680 were specifically related to basal release and were not modulated by IP3-R or AMPA-R, since neither AdA- nor AMPA-evoked releases were affected by acute administration of 100 nM CGS21680. A2AR activation inhibited astroglial glutamate transporters via inhibition of Na^+^/K^+^-ATPase activity [49]. Indeed, in the present study, the astroglial glutamate transporter blockade by PDC inhibited 100 nM CGS21680-evoked astroglial L-glutamate release.

Neither acute nor chronic administrations of 10 nM CGS21680 affected basal, AdA-, or AMPA-evoked L-glutamate release. Under the physiological condition, chronic administration of caffeine and A2AR antagonists upregulated A2AR expression [50,51]; however, chronic administration of A2AR agonist decreased A2AR expression [52,53], but increased levels of the inhibitory G-protein α-subunit [53]. In the present study, chronic administration of sub-effective concentration of CGS21680 (10 nM) did not affect expression of *Ador2a2*; however, contrary to physiological conditions, in the presence of various pathological insults, chronic A2AR activation improved A2AR hyper-expression [54,55]. This contradictory evidence suggests that understanding the clinical relevance of A2AR will require clarifying the effects of A2AR on neuro-, astroglial-, and tripartite synaptic transmissions under multiple conditions.

In the present study, both chronic administration of IFNγ (100 and 200 IU/mL) and TNFα (30 and 100 IU/mL) concentration-dependently increased basal, AdA-, and AMPA-evoked releases of L-glutamate. Chronic IFNγ (100 IU/mL) administration increased AdA-evoked L-glutamate release without affecting basal or AMPA-evoked release; however, chronic TNFα (100 IU/mL) administration increased basal and AMPA-evoked L-glutamate release without affecting AdA-evoked release. These results suggest that the stimulatory effects of IFNγ are predominantly associated with the IP3-R system rather than AMPA-R [8], whereas those of TNFα are predominantly associated with AMPA-R. Previous studies also demonstrated that chronic exposure to IFNγ increased IP3-R expression [56] without affecting AMPA-R expression [57]. Taken together with previous results, the enhanced AdA-evoked release after chronic IFNγ exposure was probably induced by increased IP3-R expression. Contrary to IFNγ, TNFα enhances function and expression of AMPA-R [58] and also increases IP3-R expression [59].

Interestingly, chronic administrations of IFNγ and TNFα decreased and increased CGS21680-induced L-glutamate release, respectively. These paradoxical action between IFNγ and TNFα on astroglial L-glutamate release associated with A2AR is dependent upon the expression of A2AR, since astroglial mRNA expression of A2AR (*Adora2a*) was decreased and increased by chronic administration of IFNγ and TNFα, respectively [60,61]. Additionally, TNFα inhibited A2AR agonist-induced desensitization of A2AR [62]. Therefore, chronic IFNγ exposure enhances astroglial transmission, by activating IP3-R-associated transmission mechanisms, but inhibits A2AR function. Contrary to IFNγ, chronic TNFα exposure enhances astroglial transmission by activating both AMPA-R-associated transmission mechanisms and A2AR function.

Activation of A2AR decreased the production of IFNγ and TNFα [35,36] and suppressed proinflammatory responses associated with IFNγ and TNFα [37,38]. Interestingly, this study demonstrated that chronic administration of sub-effective concentration of CGS21680 (10 nM) inhibited the stimulatory effects of TNFα on basal, AdA- and AMPA-evoked L-glutamate releases and prevented an increase in the *Adora2a* expression. Similar to TNFα, chronic administration of 10 nM CGS21680 inhibited the stimulatory effects of IFNγ on AdA-evoked L-glutamate release and the inhibitory effects on A2AR associated basal L-glutamate release via prevention of a decrease in the Adora2a expression by IFNγ. Thus, under the activation of IFNγ and TNFα (pathological condition), the effects of chronic activation of A2AR on astroglial release are predominant rather than that of acute activation, since acute administration of 10 nM CGS21680 did not affect astroglial L-glutamate release but chronic activation of A2AR (including a sub-clinical degree) inhibited cytokine-induced changes of astroglial transmission.

### 3.2. Effects of CBZ on Astroglial Transmission Induced by Chronic Exposure to Cytokines

CBZ is considered the A1R antagonist and A2AR agonist, since our previous microdialysis studies demonstrated that CBZ acutely increased monoamine release [32,33] by inhibiting A1R and activating A2AR [29,30], and chronic CBZ administration resulted in upregulation of A1R [26] and functional downregulation (or desensitization) of A2AR [27]. In the present study, chronic administration of therapeutic-relevant concentration of CBZ (20 μM) did not affect astroglial expression of *Adora2a* under the physiological condition but inhibited the effects of chronic administration of IFNγ and TNFα on *Adora2a* expression, similar to chronic administration of CGS21680. Acute administration of CGS21680 did not affect AdA- or AMPA-evoked L-glutamate release, whereas acute CBZ administration reduced both AdA- or AMPA-evoked L-glutamate release. Therefore, acutely inhibitory effects of CBZ on astroglial L-glutamate release are probably not modulated by A2AR associated mechanisms [17,63]. Furthermore, under the condition of glutamate transporter function, acute CBZ administration did not affect basal astroglial L-glutamate release, whereas under the glutamate transporter blockade, acute CBZ administration increased basal astroglial L-glutamate release. Contrary to CBZ, acute CGS21680 administration increased astroglial L-glutamate release, but inhibition of glutamate transporter prevented the CGS21680-evoked astroglial L-glutamate release. These discrepancies between acute administrations of CBZ and CGS21680 suggest that CBZ and CGS21680 probably activates and suppresses astroglial glutamate transporter activity. Indeed, activation of astroglial A2AR increases and inhibits astroglial L-glutamate release and astroglial glutamate transporter activity via modulation of Na^+^/K^+^ ATPases dependent pathway [49], respectively, whereas CBZ activate neuronal glutamate transporter via modulation of phosphatidylinositol 3-kinase-dependent pathway [64]. The effects of CBZ on astroglial glutamate transporter activity has not been clarified; however, the activity of astroglial glutamate transporter (GLT-1, Slc1a2) is regulated by phosphatidylinositol 3-kinase [65]. Therefore, the present results indicate the possibility that acute administration of CBZ enhances astroglial glutamate transporter. We could not conclude the effects of both CGS21680 and CBZ on basal astroglial L-glutamate release contribute to clinical action of CBZ and A2AR, since basal release induced by CGS21680 and CBZ were weak rather than those of AdA- and AMPA-evoked releases. Taken together with our previous findings, the adenosinergic action of CBZ acutely plays important roles in the neuronal transmission, but the A2AR agonistic action of CBZ acutely does not contribute to astroglial L-glutamate release.

Contrary to acute effect, chronic administration of CBZ prevented the inhibitory effects of IFNγ and the stimulatory effects of TNFα on A2AR associated astroglial L-glutamate release, similar to the effects of chronic administration of sub-effective concentration of CGS21680. Furthermore, both AdA- and AMPA-evoked L-glutamate releases, after chronic exposure to IFNγ and TNFα, were inhibited by chronic administration of both CBZ and CGS21680. Therefore, the inhibitory effects of both acute and chronic administration of CBZ on AdA- and AMPA-evoked astroglial transmitter release seem to be persistent and occur via IP3-R and AMPA-R functional modulation, whereas the A2AR agonistic action of CBZ contributes to the effects of chronic administration on astroglial transmission but does not contribute to the acute effect of CBZ.

The effects of CBZ on adenosine receptors have been underestimated by the A2AR agonistic effects of CBZ, since A1R antagonistic and A2AR agonistic effects of CBZ must be counterproductive to its anticonvulsant activity. The present study demonstrated that chronic CBZ administration suppresses the activation of astroglial glutamatergic transmission associated with IP3-R and AMPA-R induced by proinflammatory cytokines. In another line, studies emphasize that the activation of A2AR has the potential to be beneficial for the treatment of several neuropsychiatric disorders, including Niemann–Pick disease, schizophrenia and autism [66]. Therefore, in the future, comparing and determination of the effects of acute and chronic administrations of therapeutic-relevant concentration of CBZ on neuronal and astroglial A2AR functions can clarify the more detailed mechanism of clinical action of CBZ.

## 4. Materials and Methods 

### 4.1. Chemical Agents

Adenosine receptor selective agents included the A1R agonist, N6-cyclopentyladenosine (CPA, Sigma-Aldrich, St. Louis, MO, USA); A1R antagonist; 8-cyclopentyl-1,3-dipropylxanthine (DPCPX, Funakoshi, Tokyo); A2AR agonist, CGS21680 (Cosmo Bio, Tokyo, Japan); and A2AR antagonist, ZM241385 (Funakoshi) [67]. The rat recombinant interferon γ (IFNγ) and tumor necrosis factor α (TNFα) were purchased from Biolegend (San Diego, CA, USA) and R&D Systems (Minneapolis, MN, USA), respectively. Carbamazepine (CBZ) was obtained from Tokyo Chemical Industry (Tokyo, Japan). The AMPA/glutamate receptor (AMPA-R) agonist, 2-amino-3-(3-hydroxy-5-methyl-isoxazol-4-yl) propanoic acid (AMPA), inositol trisphosphate receptor (IP3-R) agonist, adenophostin-A (AdA), and non-selective glutamate transporter inhibitor, trans-4-carboxy-L-proline (PDC) were obtained from Wako (Osaka, Japan), Cosmo Bio and Funakoshi, respectively.

AdA, AMPA, PDC, IFNγ, and TNFα were dissolved in cell culture medium directly. CGS21680, DPCPX, ZM241385, and CBZ were initially dissolved at 10 mM in dimethyl sulfoxide. The final concentrations of dimethyl sulfoxide were lower than 0.2% (vol/vol). CPA was initially dissolved at 10 mM in 1N HCl.

### 4.2. Primary Astrocyte Culture

All animal care and experimental procedures described in this report complied with the Ethical Guidelines established by the Institutional Animal Care and Use Committee at Mie University (No.24-35-R1, 15/04/2014). All studies involving animals were reported in accordance with the ARRIVE guidelines for reporting experiments involving animals [68]. Pregnant Sprague-Dawley rats (SLC, Shizuoka, Japan) were housed in individual cages in air-conditioned rooms (temperature 22 ± 2 °C) with a 12 h light/dark cycle and ad libitum access to food and water. To prepare cultured astrocytes, cortical astrocytes were prepared from neonatal Sprague-Dawley rats (N = 54). The rats were sacrificed by decapitation at 0–24 h of age, and the cerebral hemispheres were removed under a dissecting microscope. The tissue was chopped into fine pieces using scissors and then triturated briefly with a micropipette. The cell suspension was filtered using 70 µm nylon mesh (BD, Franklin Lakes, NJ) and centrifuged. The pellets were re-suspended in 10 mL of Dulbecco’s modified Eagle’s medium containing 10% fetal calf serum (fDMEM), and this process was repeated three times. After 14 days of culture (DIV14), contaminating cells were removed by shaking in a standard incubator for 16 h at 200 rpm. On DIV21, astrocytes were removed from flasks by trypsinization and seeded onto a translucent PET membrane (1.0 μm) in 24-well plates (BD) directly at a density of 10^5^ cells/cm^2^ [2,8,40,69]. From DIV21–DIV28, the culture medium was changed twice a week, and various agents were added for chronic administration (7 days) (detailed methods are described under “Treatment of astrocytes and study design”). On DIV28, cultured astrocytes were washed three times with artificial cerebrospinal fluid (ACSF; NaCl 130 mM, KCl 5.4 mM, CaCl_2_ 1.8 mM, MgCl_2_ 1 mM, and glucose 5.5 mM, and buffered with 20 mM HEPES buffer to pH 7.3) (wash-out). The remaining adherent cells contained 95% GFAP-positive and A2B5-negative cells as shown using immunohistochemical staining [70].

After the wash-out, the astrocytes were incubated in ACSF (100 μL/translucent PET membrane) at 35 °C for 60 min in CO_2_ incubator (pre-treatment incubation). After the pre-treatment incubation, astrocytes were incubated in ACSF containing various agents (60 min) for acute administration and ACSF was collected for analysis. Each 100 μL ACSF sample was filtered using a Vivaspin 500–3 K filter unit (Sartorius, Goerringen, Germany). The filtered samples were freeze-dried and stored at −80 °C.

### 4.3. Treatment of Astrocytes and Study Design

At DIV21, astrocytes were incubated in fDMEM alone (control) or in fDMEM containing CGS21680 (10 nM), CBZ (20 μM), IFNγ (100 and 200 IU/mL), and TNFα (30 and 100 IU/mL) for 7 days (from DIV21 to DIV28). At DIV28, astrocytes were washed with ACSF, and then incubated in ACSF buffered with 100% O_2_ for 30 min recovery at 35 °C (wash-out). After wash-out, astrocytes were incubated in ACSF containing the same agents with chronic administration for 30 min, and ACSF was collected for analysis as basal release. After collection for basal release, astrocytes were incubated in ACSF containing the same agents with AMPA (100 μM) or AdA (1 μM) for 30 min. The study was composed of nine experimental designs. Cultured astrocytes were randomly assigned to treatment groups in each experimental design. After the experiments, to check the viability of astrocytes, the total protein level was determined using Protein Assay Reagent kit (Wako). In the present study, there were no agents affecting total protein levels in cultured astrocytes.

### 4.4. Determination of Levels of L-Glutamate 

Where possible, we sought to randomize and blind sample data. To determine the L-glutamate level, each sample was selected using an auto sampler according to a table of random numbers.

L-glutamate levels were determined by ultra-high-performance liquid chromatography (xLC3185PU, Jasco, Tokyo, Japan) with fluorescence resonance energy transfer detection (xLC3120FP, Jasco) after dual derivatization with isobutyryl-L-cysteine and o-phthalaldehyde [40,69,71]. Derivative reagent solutions were prepared by dissolving isobutyryl-L-cysteine (2 mg) and o-phthalaldehyde (2 mg) in 0.1 mL of ethanol followed by the addition of 0.9 mL of sodium borate buffer (0.2 M, pH 9.0). The automated pre-column derivatization was carried out by drawing up a 5 μL aliquot of standard or blank solution and 5 μL of derivative reagent solution and holding this mixture in reaction vials for 5 min prior to injection. The derivatized samples (5 μL) were injected using an auto sampler (xLC3059AS, Jasco). The analytical column (YMC Triat C18, particle 1.8 μm, 50 × 2.1 mm, YMC, Kyoto, Japan) was maintained at 45 °C, and the flow rate was set at 500 µL/min. A linear gradient elution was performed over 10 min with mobile phase A (0.05 M citrate buffer, pH 5.0) and B (0.05 M citrate buffer containing 30% acetonitrile and 30% methanol, pH 3.5). The excitation/emission wavelengths of fluorescence detector were set at 345/455 nm.

### 4.5. Quantitative Real-Time PCR

Astrocytes were preserved in RNAlater (Ambion, TX, USA), and total RNA was isolated with the RNeasy Micro Kit (Qiagen, Venlo, Netherlands). Briefly, total RNA was purified and reverse-transcribed into cDNA using ReverTraAce qPCR RT Master Mix with gDNA Remover (Toyobo, Osaka, Japan), according to the manufacturer’s instructions. PrimeTme real-time PCR assay kits for Ador2a2 (Rn.PT.58.35084257), Slc1a2 (Rn.PT.58.35247770), Slc1a3 (Rn.PT.58.37341518), and Gapdh (Rn.PT.39a.11180736.g) as endogenous control were purchased from Integrated DNA Technologies (Coralville, IA, USA). Real-time quantitative PCR was performed in the CFX96 (BioRad, Hercules, CA, USA). Changes in mRNA expression level were calculated after normalization to Gapdh. The ΔΔCT method provides a relative quantification ratio according to calibrator that allows statistical comparisons of gene expression among samples. Values of fold changes in the control sample versus target agents administrated samples represent averages from triplicate measurements. Changes in gene expression were reported as fold changes relative to controls.

### 4.6. Statistical Analysis

All experiments were designed with equal sample sizes (N = 6) in each group. All values were expressed as mean ± SD. Data were analyzed using the student’s T-test and one-way or two-way analysis of variance (ANOVA). When the F-value was significant (*p* < 0.05), ANOVA was followed by Tukey’s post hoc test (BellCurve for Excel, Tokyo, Japan). The data and statistical analysis comply with the recommendations on experimental design and analysis in pharmacology.

## 5. Conclusions

The present study demonstrated that both acute and chronic administrations of therapeutic-relevant concentrations of CBZ suppressed excitatory astroglial glutamatergic transmission associated with IP3-R and AMPA-R; however, A2AR agonistic action of CBZ does not contribute to acutely its inhibitory effects on astroglial transmission, but chronically plays important roles in the inhibitory effect of CBZ on astroglial transmission and proinflammatory cytokine responses. Chronic activation of A2AR activity inhibited the enhanced IP3-R and AMPA-R induced astroglial glutamatergic transmission induced by chronic exposes to IFNγ and TNFα. Therefore, the A2AR agonistic action of CBZ contributes to chronic mechanisms of CBZ against several neuropsychiatric disorders via inhibition of astroglial pathomechanisms of proinflammatory responses of IFNγ and TNFα.

## Figures and Tables

**Table 1:** Effects of CBZ and CGS21680 on Astroglial L-Glutamate Release.

	Glutamate Transporter Functional				Glutamate Transporter Blockade		
	Basal	AdA-Evoke	AMPA-Evoke	CGS-Evoke	Basal	AdA-Evoke	AMPA-Evoke
CBZ (Acute)	→	↓	↓		↑	↓	↓
CBZ (chronic)	→	↓	↓	→	↑	↓	↓
CGS (Acute)	↑	→	→		→		
CGS (Chronic)	→	→	→	→			

Effects of acute administration of therapeutic-relevant concentration of CBZ (20 μM) and effective concentration of CGS21680 (100 nM), as well as chronic administration of therapeutic-relevant concentration of CBZ (20 μM) and sub-effective concentration of CGS21680 (10 nM) on basal, 1 μM AdA-evoked, 100 μM AMPA-evoked, and 100 nM CGS21680-evoked astroglial L-glutamate releases under the conditions of astroglial glutamate transporter functional (left) and blockade (right) are indicated in Table 1. →: Did not affect, ↑: Increased, and ↓: Decreased astroglial L-glutamate release.

**Table 2:** Effects of CBZ and CGS21680 on Astroglial L-Glutamate Release after Chronic Cytokine Administration.

	After Chronic IFNγ Administraion				After Chronic TNFα Administraion			
	Basal	AdA-Evoke	AMPA-Evoke	CGS-Evoke	Basal	AdA-Evoke	AMPA-Evoke	CGS-Evoke
CBZ (Acute)	→	↓	↓		↓	↓	↓	
CBZ (chronic)	→	↓	↓	↑	↓	↓	↓	↓
CGS (Chronic)	→	↓	→	↑	↓	↓	↓	↓

Effects of acute and chronic administrations of therapeutic-relevant concentration of CBZ (20 μM) and sub-effective concentration of CGS21680 (10 nM) on basal, 1 μM AdA-evoked, 100 μM AMPA-evoked, and 100 nM CGS21680-evoked astroglial L-glutamate releases after the chronic administration of 100 IU/mL IFNγ (left) and TNFα (right) are indicated in Table 2. →: Did not affect, ↑: Increased, and ↓: Decreased astroglial L-glutamate release.

## Abbreviations

A1R	adenosine A1 receptor
A2AR	adenosine A2A receptor
ACSF	artificial cerebrospinal fluid
AdA	adenophostin A
AMPA	amino-3-(3-hydroxy-5-methyl-isoxazol-4-yl)propanoic acid
ANOVA	analysis of variance
CBZ	carbamazepine
CPA	N6-Cyclopentyladenosine
fDMEM	Dulbecco’s modified Eagle’s medium containing 10% fetal calf serum
DPCPX	8-cyclopentyl-1,3-dipropylxanthine
IFNγ	interferon γ
IP3-R	inositol trisphosphate receptor
UHPLC	ultra-high-performance liquid chromatography.
TNFα	tumor necrosis factor α
